# Smart Management Consumption in Renewable Energy Fed Ecosystems †

**DOI:** 10.3390/s19132967

**Published:** 2019-07-05

**Authors:** Francisco Javier Ferrández-Pastor, Juan Manuel García-Chamizo, Sergio Gomez-Trillo, Rafael Valdivieso-Sarabia, Mario Nieto-Hidalgo

**Affiliations:** 1 Department of Computer Technology and Computation, University of Alicante, 03690 Alicante, Spain; 2 Federación Empresas Metal Provincia Alicante (FEMPA), 03008 Alicante, Spain

**Keywords:** artificial intelligence paradigms, Internet of Things, smart grid, cloud services, embedded devices

## Abstract

Advances in embedded electronic systems, the development of new communication protocols, and the application of artificial intelligence paradigms have enabled the improvement of current automation systems of energy management. Embedded devices integrate different sensors with connectivity, computing resources, and reduced cost. Communication and cloud services increase their performance; however, there are limitations in the implementation of these technologies. If the cloud is used as the main source of services and resources, overload problems will occur. There are no models that facilitate the complete integration and interoperability in the facilities already created. This article proposes a model for the integration of smart energy management systems in new and already created facilities, using local embedded devices, Internet of Things communication protocols and services based on artificial intelligence paradigms. All services are distributed in the new smart grid network using edge and fog computing techniques. The model proposes an architecture both to be used as support for the development of smart services and for energy management control systems adapted to the installation: a group of buildings and/or houses that shares energy management and energy generation. Machine learning to predict consumption and energy generation, electric load classification, energy distribution control, and predictive maintenance are the main utilities integrated. As an experimental case, a facility that incorporates wind and solar generation is used for development and testing. Smart grid facilities, designed with artificial intelligence algorithms, implemented with Internet of Things protocols, and embedded control devices facilitate the development, cost reduction, and the integration of new services. In this work, a method to design, develop, and install smart services in self-consumption facilities is proposed. New smart services with reduced costs are installed and tested, confirming the advantages of the proposed model.

## 1. Introduction

**Figure 1:**
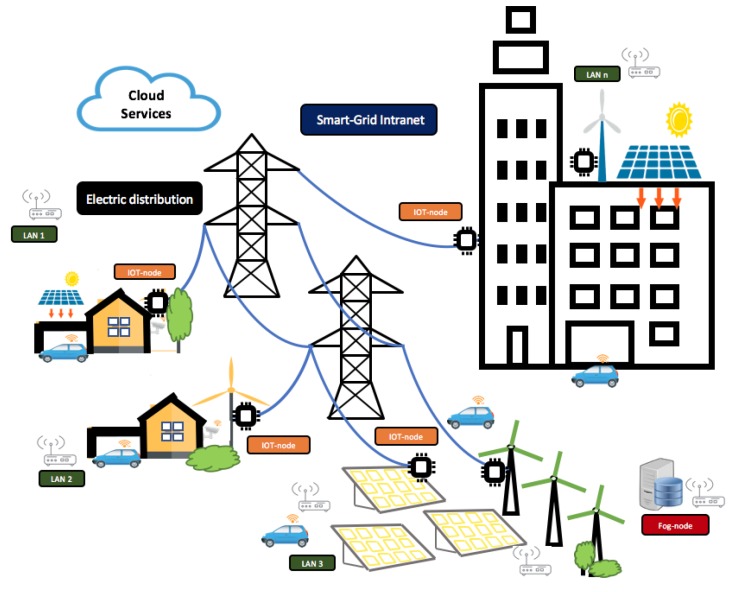
Model scenario proposed. Different sources of renewable energy, buildings, and housing are organized in a management layer (smart grid intranet). IoT nodes, smart grid layer, electric distribution control, and cloud services are the items shown.

Building and home automation systems use open communication standards and interfaces that can integrate a wide choice of different control disciplines like heating, ventilation, air conditioning, illumination safety features, and control equipment. Pre-existing buildings, however, do not usually have these advanced systems. Generally, each type of installation offers specific services: Heating Ventilation and Air Conditioning (HVAC), security cameras and sensors, comfort, user interfaces, computer asset management, etc. When an energy management system is designed, the different subsystems are related and connected through the building’s energy management system. The energy management services are specialized software. In this scenario, different manufacturers find it difficult to integrate new functionalities in facilities with different services, both for users and maintenance technicians. Automated buildings integrate software for control and data acquisition with industrial protocols and interfaces. Furthermore, integrating new services into this type of solution is not easy. These industrial developments offer cloud-connected solutions and intelligent services for energy management integrated in a vertical conception. The main purpose of operating with a vertical communication system is to control the flow of information and decision-making. Advanced processing tasks are developed at the highest levels. This model has the disadvantage of depending on the different lower levels of communication and processing. In a horizontal model, part of the services of the upper layers is implemented at lower levels, decoupling the dependency on tasks that are more optimal if they are performed in the environment where the data are generated. The state of the current technology allows transferring the services that are carried out at the high levels of a vertical architecture to the lowest levels, proposing services on a horizontal model. Advanced computer services can be implemented where the origin of the data occurs using edge and fog computing paradigms. This new scenario is deployed in Internet of Things (IoT) ecosystems and enables the design and integration of new models and services based on the smart grid paradigm. Smart environments in energy management and optimization services are the beneficiaries of these advances. This work provides a model that can be implanted using the smart grid paradigm shown in Figure 1. The objective is to optimize the management of generation and consumption, in addition to developing new control and management services for the maintenance of facilities. The contributions of this work have different levels: design an architecture hardware-software for energy management scenarios where installation, operation, and maintenance are easy to start and maintain, where energy optimization can be in facilities already developed, and economic costs are amortized in short times. A solution is proposed for small groups of facilities that can form a smart grid managed by a distributed architecture based on capture, processing and communication nodes. These nodes are of two types, according to their functionalities, which work in local communication and the cloud using IoT communication protocols.

This paper is organized as follows: Section 2 reviews related technologies, Internet of Things (IoT), Artificial Intelligence (AI) algorithms, energy management systems, and the fog and edge computing paradigm. Section 3 and Section 4 propose a model and methods to deploy the embedded hardware, IoT protocols, and AI in smart grids. Section 5 and Section 6 present the experiments conducted. Finally, Section 8 describes conclusions and future works.

## 2. Related Work

This section addresses the research lines related to energy management systems using the latest information, communication, and intelligent control technologies. The integration of the Internet of Things, artificial intelligence paradigms, embedded electronic systems, and energy management systems to optimize its use is revised.

### 2.1. Communication Protocols

The Internet network was designed in a client-server model. The client always starts the request to the server. In IoT communications, the server needs to push data to a client without the client first making a request. Software developers have come up with some techniques to overcome this challenge. New protocols adapted for this new way of working have been created and developed in recent years [1]. These protocols are used to optimize communication services. IoT needs new protocols adapted to the communication requirements. These new protocols are developed offering different options in different contexts. IoT has now a wide range of applications [2,3,4,5]. One of the most widely-used protocols, both at the local network and the Internet network level, is the Message Queue Telemetry Transport protocol (MQTT). In this work, this is the protocol that is used as a communication channel between the different nodes. MQTT [6] is a Machine-to-Machine (M2M)/“Internet of Things” connectivity protocol. It was designed as an extremely lightweight publish/subscribe messaging transport. It is useful for connections with remote locations where a small code footprint is required and/or network bandwidth is limited.

### 2.2. Energy Management Systems

The Energy Management Systems (EMS) can be classified into vertical and horizontal applications. On the one hand, vertical EMS are focused on particular applications [7,8], homes [9,10], buildings [11,12], data centers [13,14], smart cities [15,16], etc. Vertical EMS exploit their own features in order to optimize energy generation and consumption, but are not suitable for other applications. On the other hand, horizontal EMS are model-driven proposals, designed in order to be used in different applications or scenarios. Traditionally, three types of control for energy flow management are used: centralized, distributed, and hybrid [17]. A decentralized energy management for a polygeneration microgrid topology was described in [18]. It was based on multi-agent systems and fuzzy cognitive maps. A multi-agent system for a decentralized control architecture for distributed generation was defined in [19]. These multiagent systems were simulated, but did not consider the hardware infrastructure required to be deployed in a real scenario. Recent proposals related to EMS were based on cloud, fog, and edge computing paradigms. An energy management system as a service was proposed in [20] over the fog computing paradigm. The main features of this proposal were derived from the fog computing paradigm: interoperability, scalability, adaptability, and connectivity. To achieve these features, it used: low-cost/-power devices, open software/hardware infrastructure, and energy management software developed as a SOAPweb service. They presented two case study: home energy management and microgrid energy management. This proposal was more appropriate for small facilities than large facilities, due to the advantages of the fog computing paradigm not being suitable for large facilities, because it requires a large number of fog computing nodes. A fog-based architecture for EMS was proposed in [21]. The architecture was composed of three layers: home gateways, fog nodes, and cloud servers. Home gateways acted as collectors of energy consumption and the user interface. Fog nodes were the retail energy market server, which provided energy services to the end-users. Cloud servers were for data storage and high computing power. This proposal did not consider load management in order to reduce power consumption. There are related algorithms for subsystems of EMS. An algorithm for EMS based on multi-layer ant colony optimization for microgrid applications was presented in [22]. The algorithm met the required load demand with minimum cost in a local energy market under five-minute real-time scheduling. The energy load forecasting methodology based on deep neural networks was proposed in [23]. The algorithms tested were variants of Long Short Term Memory (LSTM). The horizontal EMS proposals analyzed were based on different paradigms like multi-agent systems, cloud, fog, and edge computing, according to the aim of each one. Most of the proposals were not general enough and did not include features like predictive consumption/generation, load management, load classification, and predictive maintenance. The architecture proposed offered smart services based on machine learning algorithms, and it was at different layers in order to manage building facilities.

### 2.3. Optimization Systems

In addition to systems based on communication protocols or architectures for energy management systems’ development, there were works that focused on optimizing the use of energy. In this line, the interconnection between smart grids was studied to optimize the use of energy. In [24], the energy trade for interconnected microgrids was analyzed. An optimization model based on algorithms was proposed as the contribution. Optimizing the economic cost is the main objective of this type of work. In [25], a cooperative planning of renewable generations for interconnected microgrids was proposed. Other optimization systems were [26,27], which demonstrated the interest during the last few years in this type of approach, where the frameworks and software systems for the interconnection between different microgrids were analyzed.

### 2.4. Findings

The novel aspects of this work are based on the integration of different technologies. The model proposes a method that is capable of making interoperable low-cost hardware technologies with complex artificial intelligence algorithms, using communication protocols that are still emerging. The methods used allow the use of different technologies, as they develop. It can be installed in already built or newly-designed facilities. It can be used in smart grid designs with or without an electrical grid connection. It uses different paradigms of artificial intelligence in a simple architecture based on two node types (edge node and fog node) with flexible functionality with capacities adapted to the proposed objectives. The model used non-proprietary hardware and software and did not depend on any specific platform in the cloud.

In this context, similar systems have as a preference the minimization of the cost of energy using the rates and prices of the energy unit. The proposed model in this work is based on optimization so that the minimum difference between generation and consumption is achieved. This objective reduces the need to use batteries in connected installations. In this case, the cost of installation and maintenance is optimized. In systems where prediction or classification algorithms are applied, the processes are performed for computers and servers. In this work, these algorithms are designed and implemented near physical systems and integrated devices. Regarding the percentages of success in the classification or prediction, the improvements are similar to other AI systems. In systems where prediction or classification algorithms are applied, the processes are performed on computers and servers. In this work, these algorithms are designed and implemented near physical systems and integrated devices. Regarding the percentages of success in the classification or prediction, no significant improvements are achieved since this is not the objective.

## 3. Architecture Model

**Figure 2:**
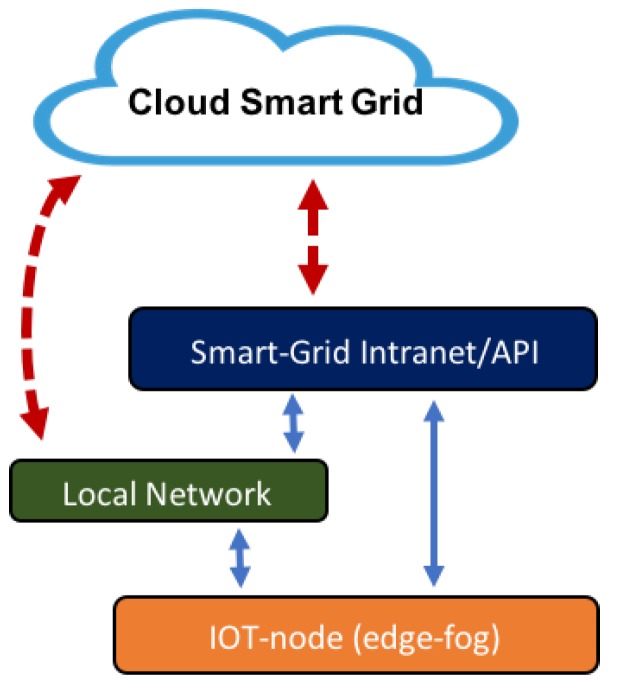
Model layer architecture with the main elements and their relationship. There are two node types: edge and fog. Each facility (building, solar generation, etc.) can have its own local network. All the nodes and all local networks are related to the energy (smart grid) and cloud management layers.

The model proposes a layer-based architecture. Each power installation (building, house, renewable station, etc.) has its own local network, which is integrated into the model according to the architecture shown in Figure 2. All subsystems offer necessary services in management operations. Each subsystem acts in this scenario and can be interoperable with the other subsystems. Horizontal and vertical algorithms are designed and implemented at different levels. Two IoT nodes types (edge and fog) are defined. The edge node is the device closest to the control and operation. This node can implement simple artificial intelligence algorithms that act in the local area where it is installed. It communicates through standard protocols with the rest of the layers. The edge node implements mainly horizontal control algorithms and also vertical communication to other levels. They are designed so that they can function autonomously, decoupling their dependence. The fog node has greater computational capacity than the edge; it can store data and perform the analysis of the operation of energy management, using artificial intelligence algorithms and interfaces that take data globally. Its scope is not linked to the local environment as the edge node; it is greater. It implements vertical algorithms because it receives and propagates services to other levels.

The main components of the proposed architecture are defined below.

### 3.1. IoT Nodes: Edge and Fog Levels

Edge and fog IoT nodes allow designing different types of solutions and services. As described above, an edge node device controls the installation locally, that is it manages the sensors, actuators, and interfaces of the environment in which it is installed. According to this definition, the node has control algorithms with support tasks based on artificial intelligence. An edge node connected in a building can define consumption patterns, classify electrical connections, predict consumption, and detect events for predictive maintenance services. All this is in the environment of the building that it controls. The model designs smart grids on groups of houses (already built or not) and generation units that form flexible groups managed by a set of services. These services can be installed on hardware nodes near the installation (edge node) or on a server that manages the network formed (fog node).

- An edge node is a programmable controller with resources to capture sensor data, with minimum capacity to store data, process basic algorithms with mathematical models for classification and prediction, and with resources to communicate data in a local intranet. It is the device closest to the control and operation units of the facilities.
- A fog node has a processing and storage architecture with greater capacity than the edge node. It corresponds to a storage and services server that also develops classification and prediction applications at the smart grid level. It can be a PC or a server. Its location does not depend on the place where the control facilities are located.

#### 3.1.1. Edge Nodes

In the field of energy management, the **edge node** can perform some of the following tasks:

- **Power consumption and generation data capture**: If the node is installed in a consumer electrical panel, it obtains electrical data related to the devices’ connection and disconnection. The electrical data can be the intensity, voltage, active and reactive power, power factor, harmonics, and all types of data that can be used to understand the operation of the installation. If the node is installed at a generation point, it obtains electrical data from the energy produced, just as in the case of consumption.
- **Other data type capture**: To design prediction or regression models, sometimes the nodes capture another type of data of interest as it could be environmental data: temperature, humidity, radiation, weather conditions, or other types of data related to the operation that can be used by the artificial intelligence models.
- **Filtering and data preprocessing**: The captured data must be revised to avoid erroneous entries. They are also normalized to be used as inputs to the management algorithms. Sometimes, the data are preprocessed and filtered with some mathematical transformation (Fourier, wavelets, vector transformation) to obtain information or to reduce the amount of relevant data.
- **Actuators’ control actions**: For consumer installations, the edge node installs circuit connection and disconnection actuators. In this way, services that control and optimize the distribution and use the available energy are integrated. These services can be installed in already built facilities or in new construction projects. For generation systems, the node switches and directs production to the points where it produces the greatest benefits and optimizes its use. The management of storage of energy and use without the need to store comprises two critical tasks to be solved by this type of node in the control function.
- **Classification models**: At the local level, the edge node can implement algorithms based on classification and detection models. In consumer facilities, the node uses models trained with connection data during the learning phase to classify different types of consumers.
- **Prediction models**: At the local level, the edge node can implement algorithms based on prediction models. In consumer facilities, the edge node uses past consumption data to predict future consumption. In generation facilities, the node obtains meteorological data and past generation data to predict the generation of the installation in the next hours
- **Communication processes**: The edge nodes communicate with the different levels and layers using IoT protocols. A node can send the capture and classification data to the smart grid management layer, transmit consumption information to the local network, or the status of the actuators to services installed in the cloud.
- **User interfaces**: At the local level, the edge node can implement different user interfaces. Web pages, local dashboards, and smartphones interaction are some examples.

**Figure 3:**
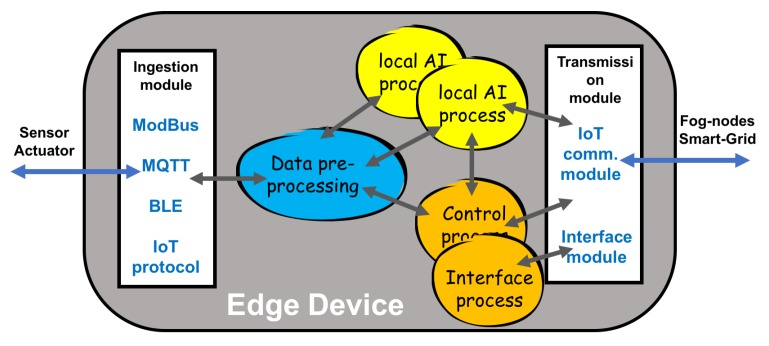
Relationship between the modules in an edge node according to the established requirements.

Figure 3 shows the edge node modules (input, output, processing, interfaces, and communication processes) and their relationship.

#### 3.1.2. Fog Nodes

The fog node introduces new capabilities and scopes of application, in relation to the previous node. It implements the same communication protocols as the edge node, but with different functions. A **fog node** can perform some of the following tasks:

- **Learning**: In these nodes, prediction and classification models are designed and tested to be installed in the different network nodes. The fog nodes participate in the learning phase, where the dataset of the variables that intervene in the energy management processes of the installation are analyzed.
- **Analysis**: The data transmitted by the edge nodes in the operation processes are analyzed by applications developed in the fog nodes. The results of the classifications and the predictions allow evaluating if the algorithms implemented are working at the established levels.
- **Data storage**: The data and relevant information of the smart grid, supplied by the edge nodes, by the internal processing itself or by the services implemented in the cloud, are stored in these nodes in different types of formats, according to the use (databases, files, links, etc.). If part of the information is saved in the cloud, those nodes will only keep the necessary data to be able to work without having to depend on Internet connections.
- **Data capture**: In the same way as edge nodes, these nodes can obtain data and datasets needed to perform network management actions. These nodes capture data that can be used by other devices in the network, such as weather forecast data or energy cost data.
- **Artificial intelligence algorithms**: Algorithms for classification, detection, prediction, or predictive maintenance are designed and implemented for services used in different nodes. Therefore, these algorithms can be applied to all nodes and installations of the smart grid.
- **Interfaces**: In the fog nodes, the Human–Machine Interface (HMI) and Machine-to-Machine interfaces (M2M) are implemented at the smart grid level. Different devices become interoperable using the interfaces developed in these nodes.

**Figure 4:**
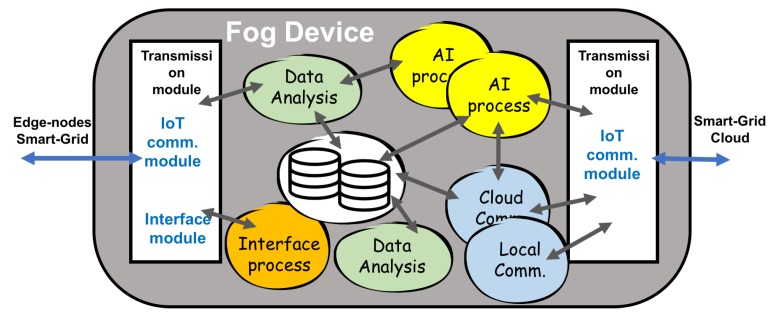
Relationship between the modules in a fog node device according to the established requirements.

Figure 4 shows the fog node modules (input, output, processing, data storage, interfaces, and communication processes) and their relationship.

### 3.2. Local Smart-Grid Intranet: Vertical Services

The smart grid layer manages the different nodes and is used as an interface to the cloud services. The tasks it performs are:

- API management support
- Security features
- Maintenance functions
- Interoperability implementation between nodes
- Cloud service support

In this work, this level develops interoperability tasks between the different nodes and the interface with the cloud.

### 3.3. Cloud Smart Grid: Communication, Interfaces, and Big Data Services

The cloud provides a set of resources to optimize and make interoperability global. The smart grid can connect to other smart grids using cloud services. The cloud computing paradigm provides, among others services, low-cost storage, accessibility, scalability, and interoperability. The cloud is used in a set of utility services. The main services are:

- **Treatment of a large amount of IoT devicesand Big Data**: Smart grids of all sizes collect enormous quantities of complex, fast-moving data that contain value that may give them a competitive edge or lead to better decisions. The cloud is a good option to assist with Big Data workloads. This is because the cloud provides a centralized platform with access to powerful computing infrastructure and inexpensive storage at a relatively low cost.
- **Cloud data analytics**: Numerous providers in the cloud (AWS [28], Google [29], Ubidots [30], and Microsoft [31]) are beginning to offer higher performance storage and analysis by artificial intelligence paradigms using using their platforms. The model can use these resources to process data at this level. Cloud providers already have the technologies in place to deliver their own powerful AI infrastructure to energy data analysis.
- **Dark data use**: Gartner defines dark data [32] as the information assets that organizations collect, process, and store during regular business activities, but generally fail to use for other purposes (for example, analytics, business relationships, and direct monetizing). Thus, organizations often retain dark data for compliance purposes only. Storing and securing data typically incur more expense (and sometimes greater risk) than value. In the electric management model, dark data extraction tools that can identify garbage information versus valuable information must be used to optimize the cloud.
- **App development**: Cloud support offers back-end application development platforms for monitoring applications, Human–Machine Interfaces (HMI), communication, and intelligent control.
- **Event management**: Electrical facilities require constant monitoring, where scheduled events of interest (failures, non-normal operation, programmed levels reached, etc.) must be detected and communicated. Cloud platforms allow this type of use.
- **Cloud Application Programming Interface (Cloud API)**: The API enables the development of applications and services used for the provisioning of cloud hardware, software, and platforms. The cloud API serves as a gateway or interface that provides direct and indirect cloud infrastructure and software services. Local and cloud smart grids use the API cloud as a Software as a Service (SaaS) (software or application provision connectivity and interaction with a software suite).

## 4. Computing Model

Electric management in smart grids (formed by buildings, homes, and generation facilities) must analyze the different subsystems (consumers, distributors, and producers). These subsystems, in automated facilities, are controlled by specialized control technologies. In non-automated facilities, these services do not exist, and the subsystems are controlled electronically and electrically. In both cases, the aim of the model proposed in this work is to create new utilities and solutions to the requirements of energy management for groups of homes and buildings forming a smart grid. The model uses already installed facilities and incorporates new ones. The smart grid can be developed in new or already built facilities.

**Figure 5:**
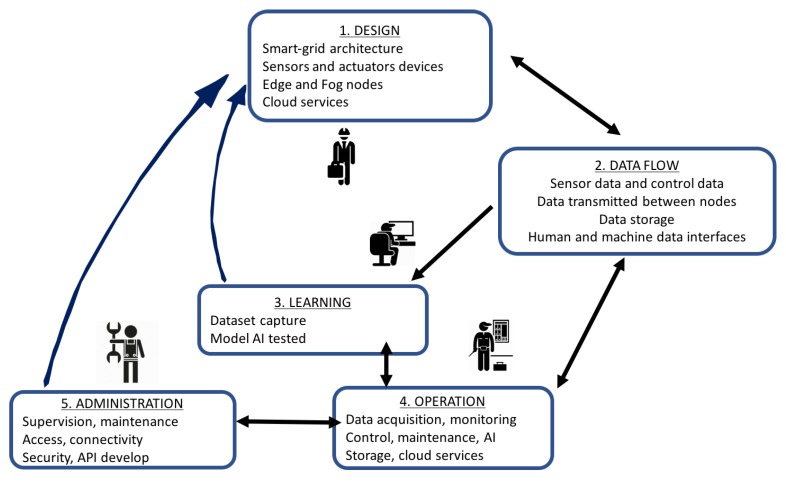
Computing model.

In the initial phase of the smart grid design, the set of requirements that the electric manager should follow should be established. These requirements are transferred to the computational model, which integrates the intelligent services into the necessary infrastructure. Depending on the services, the computational model will require a set of hardware and software elements. All this process is described in the following phases proposed in the model (Figure 5).

The model develops the following phases:

1. **Design**: The objectives and requirements are defined. Hardware, software, and communication resources (hardware, communication and control protocols, software services, etc.) necessary for the smart grid development are treated in this phase. The main elements are:
  - New services, improvements, and optimization.
  - Sensors and actuators that are necessary for installation. The type, location, functionalities, and associated edge device.
  - Edge nodes. The type, capacities, algorithms installed, place of installation, relationship with sensors and actuators, type of communication with the smart grid, and other nodes.
  - Fog nodes. Similar to the edge node, expanding with the type of communication and relationship with storage services, network management, and cloud services.
  - Smart grid architecture. Management and maintenance services used.
  - Cloud platform type. Services, communication protocols, and applications. Front-end and back-end design.
2. **Data flow**: The electrical data must be captured, filtered, processed, and transmitted between the different nodes of the network. In this phase, all the processes related to these tasks are analyzed:
  - Sensors’ data captured by power meters and other related sensors (meteorological, environmental, state of the machines, etc.).
  - Data filtering and normalization.
  - Data transmitted from the edge node to the fog node and to the smart grid management services.
  - Data storage.
  - Data transmitted from the fog nodes to the edge node and to the management layer.
  - Data transmitted to the cloud and received from cloud services.
  - Control data. Data sent to the distributors and actuators.
  - Human–machine and machine–machine interfaces.
3. **Learning**: Vertical procedures are used to define the processing and communication services, as well as the necessary horizontal algorithms in each of the nodes. Machine learning patterns and artificial intelligence models are designed in this phase. To implement detection, classification, and prediction processes, it is necessary to address a learning phase using the data captured and treated. This learning phase depends on the type of application that must be solved.
  - In power load classification, the learning process develops a pattern detection model. In this case, the learning process creates a classification model after capturing the patterns that define the type of power loads.
  - In a generation prediction process, the learning process must create a regression model. In the learning process, the task of creating the regression model is performed, which depends on the type of installation.
  - In a predictive maintenance process, a model based on rules that detect singular events must be established.
  - Other processes based on artificial intelligence paradigms are trained in this initial phase of learning.
4. **Operation**: Algorithms are installed in embedded devices in horizontal solutions to control local facilities. Control and communication services are developed to transmit data to horizontal and vertical layers. In this phase, after the necessary learning, the control, classification, and prediction algorithms are installed in each of the nodes defined in previous phases. The algorithms are executed, and the installation starts the operation process.
  - Capture and filtering processes.
  - Reactive control processes, based on rules.
  - Supervision processes, classification, detection, or prediction processes, based on artificial intelligence models, communication processes, based on IoT protocols, processes of data storage, through the use of specialized databases, access and interoperability interfaces based on adapted programming paradigms.
  - Storage.
  - Cloud services.
5. **Supervision, management, and maintenance**: Vertical algorithms show the results of operating processes using HMI interfaces. The smart grid must be maintained and managed: different elements like data, node devices, or electric actuators are analyzed. The data network is administered with specialized computer resources that guarantee operation at this level.

## 5. Experimental Design and Results Analysis

The model introduces a layer architecture, the set of processes that can be installed at each level and the devices needed to design and develop a local smart grid. Experimentally, three types of houses were used that had solar generation installed. In these facilities, a smart grid was designed to compare the advantages using the services proposed on the smart grid.

**Figure 6:**
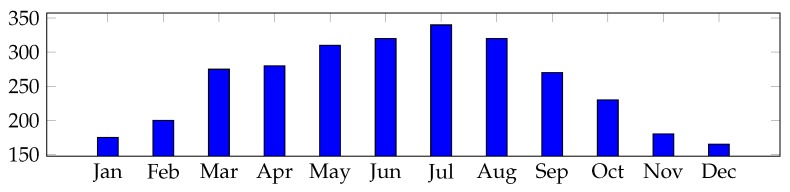
Power generation estimate: average Spanish peninsular in kWh (vertical axis) for each month considering a 1500-W module installed.

**Figure 7:**
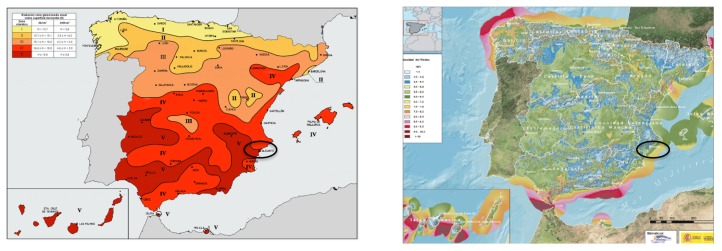
Wind and solar resource in the installation area.

The facilities used in each house were photovoltaic modules of 500, 1000 W or 1500 W. Energy captured in one day was 4950 Wh in winter and 10,550 Wh in summer for 1500 W. For the production estimate, the average Spanish peninsular radiation was taken into account, including a percentage of system losses and a capturewith a minimum of 3 h of peak sun for winter, while in summer, 7 h of sunshine were taken (Figure 6 and Figure 7).

Each house or building had different types of power consumption. The graphs in the figure show the energy consumption patterns (blue lines) with which the experimental work was conducted. On each chart is included the renewable power produced (green lines).

**Figure 8:**
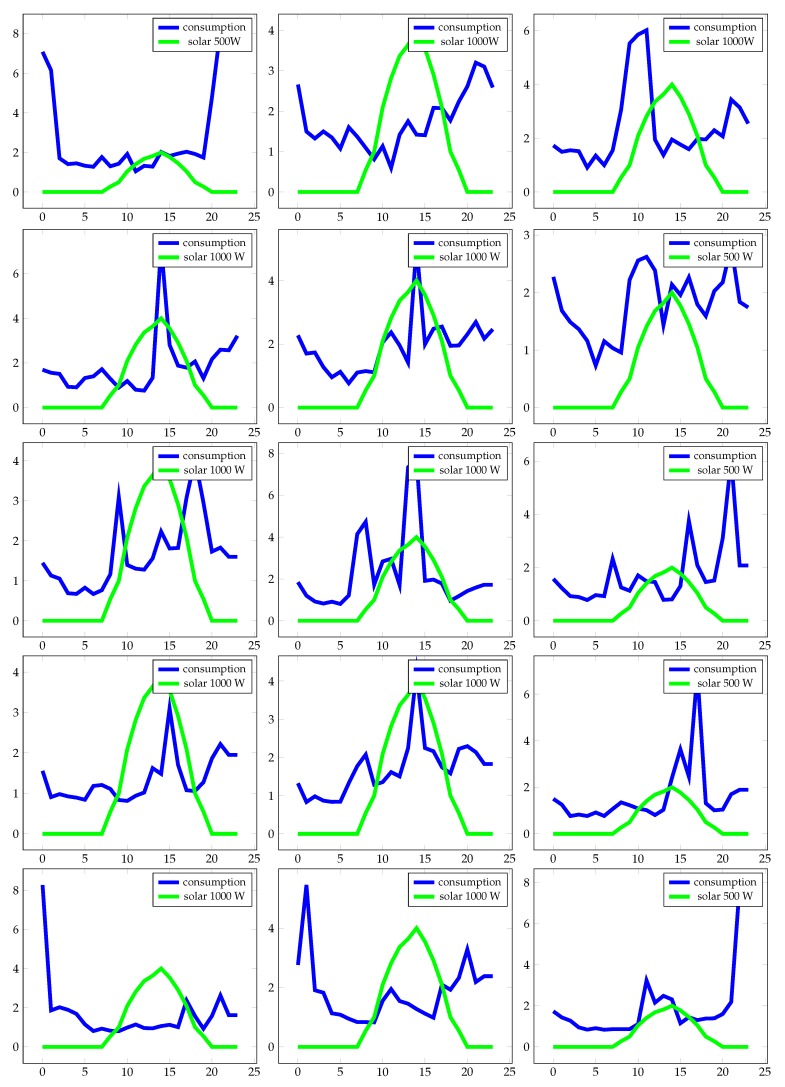
Subfigures represent power consumption patterns during 15 days (blue line) and power generation (green line) in different houses. The X axis shows the time of day (in hours) and the Y axis the average electric current (amps) during the indicated time period.

In Figure 8, different patterns in power consumption and power generation show that there was generated energy that was not used at the time of production. This occurred when the energy generated was greater (green lines) than the energy produced (blue lines). Under these conditions, the system can store or simply not use energy. If it is stored, it requires a battery installation and an electronic system that controls its loading and unloading. Batteries in these cases are necessary, although there is a decrease in performance and an increase in the cost of the installation and its maintenance. Another important feature is the use of energy in each home: if there are consumption patterns, the inhabitants can change habits, be they outside seasons or other types of variations that modify the consumption patterns. When there is a significant variation, the installation may degrade in optimization and quality.

For all the reasons indicated below, a smart grid facility using the model proposed will be able to reuse power generation to optimize and provide value-added services in power management. In this work, a smart grid is designed using consumption and generation patterns obtained through the capture and monitoring of consumption and power generation data. The model develops the phases: smart grid design, data flow, learning, and operation.

### 5.1. Smart Grid Design

**Figure 9:**
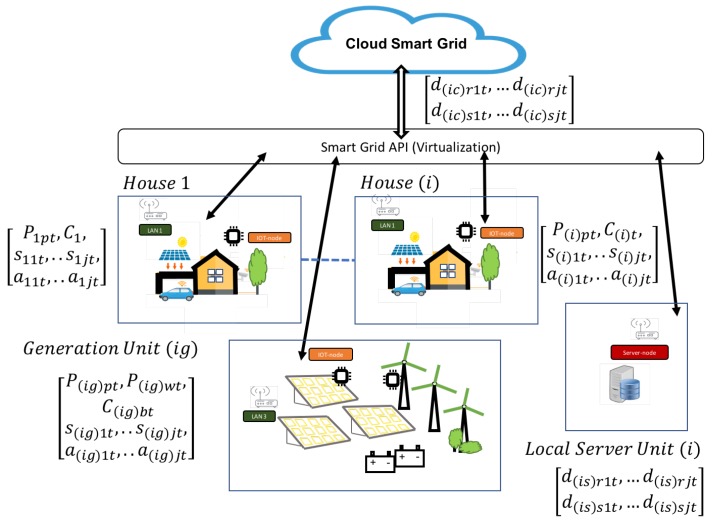
Model design: architecture diagram. Legend: $P_{(i)pt}⟶$ photovoltaic-generated power in house (i) in time *t*, ∀i=1,2,…nwithn=house, $C_{(i)t}⟶$ power consumed in house (i) in time *t*, ∀i=1,2,…nwithn=house, $I_{(i)t}⟶$ electric current consumed in house (i) in time *t*, ∀i=1,2,…nwithn=house, $s_{(i)jt}⟶$ sensor data *j* in house (i) in time *t*, ∀i=1,2,…nwithn=house, $a_{(i)jt}⟶$ actuator data *j* in house (i) in time *t*, ∀i=1,2,…nwithn=house, $P_{(ig)pt}⟶$ photovoltaic-generated power *j* in renewable module (ig) in time *t*, ∀i=1,2,…nwithn=renewableunit, $P_{(ig)wt}⟶$ wind-generated power *j* in renewable module (ig) in time *t*, ∀i=1,2,…nwithn=renewableunit, $C_{(ig)wt}⟶$ battery charge status *j* in renewable module (ig) in time *t*, ∀i=1,2,…nwithn=renewableunit, $s_{(ig)jt}⟶$ sensor data *j* in renewable module (ig) in time *t*, ∀i=1,2,…nwithn=renewableunit, $a_{(ig)jt}⟶$ actuator data *j* in renewable module (ig) in time *t*, ∀i=1,2,…nwithn=renewableunitnumber, $d_{(is)rjt}⟶$ data rj received in server (is) in time *t*, ∀i=1,2,…nwithn=serverunit, $d_{(is)sjt}⟶$ data sj sent by server (is) in time *t*, ∀i=1,2,…nwithn=serverunit, $d_{(ic)rjt}⟶$ data rj received in cloud platform (ic) in time *t*, ∀i=1,2,…nwithn=cloudplatformunit, $d_{(ic)sjt}⟶$ data sj sent by cloud platform (ic) in time *t*, ∀i=1,2,…nwithn=cloudplatformunit.

The smart grid uses photovoltaic panels already installed in the houses and proposes the predictive control of some home electrical devices that can be automated, such as home appliances, swimming pool purification engines, water tank heating, and other programmable devices. To optimize the renewable resources, a power generation unit that uses wind and photovoltaic energy is added to the smart grid (Figure 9).

### 5.2. Operation Optimization

The target is to determine the optimal operating scenario of the Consumers (C) units in combination with the renewable system (P). The model can propose different optimization functions. In this first approach, the cost of energy is not considered; it is intended to balance production and generation through two levels of optimization: first in each house and, then, the set of network facilities.

Each house (i) has an objective function:

(1) $$F_{(i)t}=C_{(i)t}-P_{(i)pt}-P_{(i)wt}+\sum_{j=1}^{k}Ca_{(i)jt}.A_{(i)jt}\;\;\forall i=1,2,\ldots n\;with\;n=house$$

The variables are:

$F_{\left(i\right)}⟶$ the function objective in house (i) in time *t*, ∀i=1,2,…nwithn=house.
$Ca_{(i)jt}⟶$ power load controlled by programmable actuator *j* in house (i) in time *t*, ∀i=1,2,…n∀j=1,2,…kwithn=house.
$A_{(i)jt}$ is one if actuator $a_{(i)jt}$ is connected, in time *t*.
$A_{(i)jt}$ is zero if actuator $a_{(i)jt}$ is not connected, in time *t*.

For each house, the objective function must be minimized. The objective is to try to make sure that all the energy generated is consumed, in the period of time established, in the house where it is generated. To minimize the function, an algorithm is used that decides the number of programmable loads $Ca_{(i)jt}$ that must be connected to balance consumption with generation(Algorithm 1).

**Algorithm 1** Minimize function $F_{(i)t}$ in house *i* (*t* = 1 h). **if** ($F_{(i)t}<0$) **then**  **while** ($F_{(i)t}<0$) **do**   connectnewprogrammable(**j**)loadinhouse(**i**$)\to A_{(i)jt}=1$   **if** (allload(j)isalreadyconnected) **then**    break   **end if**  **end while** **end if**

This process is made with a time period of 1 h, as the unit of time taken as reference in this study. Each hour, the processing node of each house predicts consumption and generation and programs the connection or disconnection of programmable loads (Algorithm 2). The smart grid has the next objective function:

(2) $$F_{smt}=\sum_{i=1}^{n}F_{(i)t}-\sum_{i=1}^{n}P_{(ig)pt}-\sum_{i=1}^{n}P_{(ig)wt}+\sum_{i=1}^{n}\sum_{j=1}^{k}C^{\prime }a_{(i)jt}.A_{(i)jt}$$

The variables are:

$C^{\prime }a_{(i)jt}⟶$ power load controlled by programmable actuator *j* in house (i), not activated in $F_{(i)t}$, in time *t*,∀i=1,2,…n.
$A_{(i)jt}$ is one if actuator $a_{(i)jt}$ is connected, in time *t*
$A_{(i)jt}$ is zero if actuator $a_{(i)jt}$ is not connected, in time *t*.

**Algorithm 2** Minimize function $F_{smt}$ in the smart grid (*t* = 1 h). **if** ($F_{smt}<0$) **then**  **while** ($F_{smt}<0$) **do**   connectnewprogrammable(**j**$)\;load\to A_{(i)jt}=1$   **if** (allload(i)jisalreadyconnected) **then**    break   **end if**  **end while** **end if**

The sensors, devices, and nodes shown in Table 1 are:

- Sensors and actuators:
  – Power meter and Current Transformer (CT) installed on the main power panels and connected to the edge node.
  – Ambient sensors (temperature, humidity, etc.) connected to the edge node.
  – Control relay installed on power panels already installed or new ones and connected to the edge node.
- Edge nodes:
  – Embedded devices installed near the operation points where they act.
  – An edge node is a device with a monoprocess with basic data processing (capture, filtering, transmission) or a device with a multi-process capability integrating algorithms for detection, classification, or prediction of the data captured by the node.
  – The node can store small amounts of data and receive data from another level of the architecture (weather forecast, environmental data, ON-OFF operation, etc.).
- Fog nodes:
  – In these nodes, different services are installed (web servers, databases, API functions, HMI interfaces) that manage the smart grid.
  – Receive and send data from the edge node and cloud services
  – Send requests to the control nodes, allowing the interoperability of the different subsystems
  – Develop the application programming interface functions.
  – The fog node needs a computing capacity with the ability to install servers and store data in specialized databases (network-attached storage); in addition, computing capacity to process IA algorithms with data that come from all the lower level nodes and higher level cloud services.

The services proposed are:

1. Monitoring: local interfaces and cloud dashboard.
2. Learning resources: AI models’ development.
3. Prediction algorithms: consumption and generation.
4. Power distribution optimization: ON-OFF automated control of electric charges.

### 5.3. Data Flow

**Figure 10:**
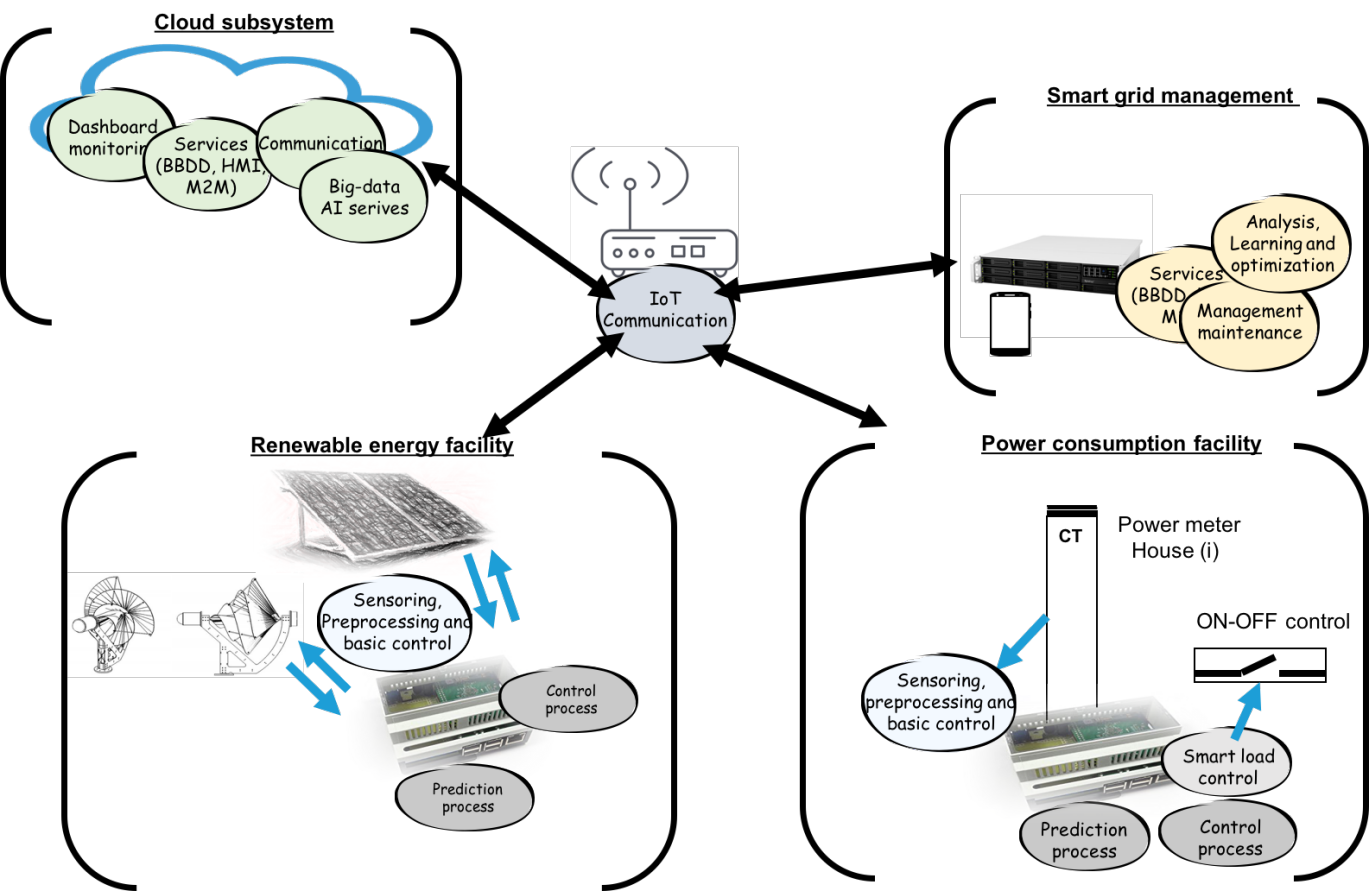
Subsystems in the data flow analysis: power consumption, renewable energy, smart grid management, cloud subsystems, and IoT communication.

Sensors, actuators, and programmable devices were deployed in the model at three levels. The experimental work was carried out in houses already built, where the devices captured and treated the data. The flow starts with the data capture to know both the consumption load curve and the power generation. The smart grid had different sensors sources and control data, processing algorithms that make decisions to optimize, communicate, and control, and needed subsystems installed for management, maintenance, analysis, and supervision. In general, the main processes involved are reflected in Figure 10. Four subsystems were established in the determination of the data flow.

- Consumption subsystem, where the sensors’, actuators’, and controllers’ data in consumption facilities were treated.
- Renewable energy subsystem, in the same way as in the previous case, but for the generation devices.
- Network subsystem, a central subsystem that managed the operation of the network and collected all the data of the different subsystems interoperable. The data of all vertical applications were processed and communicated in this subsystem.
- Cloud subsystem, reflecting the set of data sent and received from cloud services.

In the experimental work, different consumption patterns were captured. The type of connection that detected and classified the installation depended on the decision-making in the design and first captures of the sensors. There was therefore an initial phase of capturing information from the sensors to develop the necessary datasets. The houses had installed power of 3.3–9 KW. Each house had an individual photovoltaic facility of 500 or 1000 W. The installed generation unit had a wind power subsystem of 1500 W, a photovoltaic subsystem of 500 W, and a battery facility with 2500 Wh/day of capacity. The analysis of the smart grid was based on considering that the energy generated in each house could be shared with the common energy consumption network. In each house, ON-OFF actuators were installed to automate the control of loads using smart control algorithms. The automated loads, controlled in each house, could reach consumptions between 500 and 1000 W. The communication protocols used were based on producer-consumer models (Message Queuing Telemetry Transport (MQTT)) for sensors’ and actuators’ data and a client-server model (Hypertext Transfer Protocol Secure (HTTPS)) for management, interfaces, storage, and maintenance data.

### 5.4. Learning

Devices, nodes, and data flow offered support resources to design different AI services. The learning processes started with the data obtained by the different sensors and nodes installed. From the capture of consumption data, electricity generation data, along with other sensor data such as climate prediction or environmental conditions could initiate different learning processes. These processes depended on the services designed. In this experiment, the learning processes were:

- A pattern recognition subsystem to develop a connection detection and load type.
- A model to predict the level of renewable energy generated and the consumption load throughout the day.
- Automatic control rules of decision-making for the distribution of the load.

#### 5.4.1. Learning Processing: Connection Classifier

**Figure 11:**
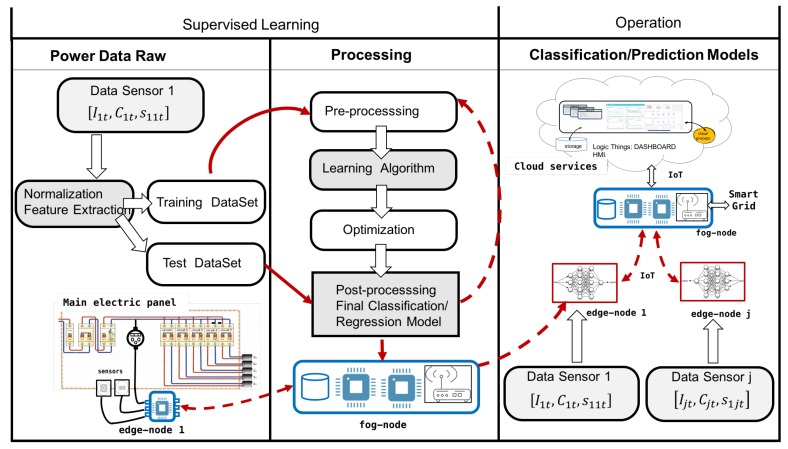
Learning and operation process in classification and load prediction.

In each house or renewable facility, the power sensors were the main elements to capture data. Considering each of the subsystems of Figure 11, training and test dataset were captured to analyze and create classifier systems.

**Figure 12:**
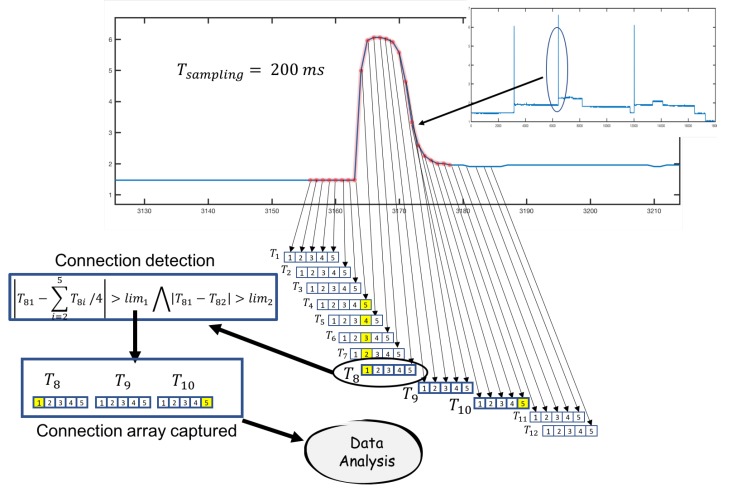
Load connection detection example. In the $T_{8}$ subarray, a connection is captured.

These datasets were formed by the first current data that were captured when a connection of an electrical load occurred. The main variable used was electric current ($I_{it}$). The time for sampling was every 200 ms, so the first data captured when a connection was detected (data array) characterized the type of load. For this process, a connection capture algorithm was installed in the edge node connected to the electrical data sensor. The array had a size of 15 values, that is lines of this size were captured and analyzed. Figure 12 shows the process of detecting and capturing a load connection. As was observed, subarrays of five values were taken, on which a first calculation was made to detect the connection. Two limit values were set for this operation ($lim_{1}$ and $lim_{2}$), indicated in the figure. If the calculation performed on the subarray detected the connection, the algorithm captured the data in the arrays $T_{8}$, $T_{9}$, and $T_{10}$ shown in Figure 12 to compose the final array with 15 data.

A formula was proposed to detect the connections from a certain level of load, according to the limits set.

If the condition on a subarray $\left(I_{1t},I_{2t},I_{3t},I_{4t},I_{5t}\right)$ of current values captured with a sampling time of 200 ms, in time *t*:

$$\left|I_{1t}-\left(\sum_{i=2}^{5}I_{it}\right)/4\right|>lim_{1}\wedge \left|I_{1t}-I_{2t}\right|>lim_{2}$$

was fulfilled, then a new device was connected in the facility. When a new load was detected, it went through a learning or operation process.

The frame (array) captured was adapted taking as reference the initial value:

$$\begin{array}{c} Data\;captured\to \left[I_{1},I_{2},\ldots ,I_{15}\right]\\ Data\;adapted\to \left[\left(I_{1}-I_{1}\right),\left(I_{2}-I_{1}\right),\ldots ,\left(I_{15}-I_{1}\right)\right] \end{array}$$

A wavelet transform process was used to normalize and obtain the feature extraction. Wavelet transform allows dimensionality reduction. The wavelet power spectrum using the Haar wavelet has the potential to identify informative features [35]. In the experimental work, a clustering and feature selection method for classification based on the Haar wavelet power spectrum was used. The local wavelet power spectrum at a particular decomposition level was calculated by summing up the squares of wavelet coefficients at that level. For a set of wavelet coefficients $C_{j,k}$ where *j* is the level of decomposition and *k* is the order of the coefficient, the wavelet power spectrum is given below:

$$Data\;adapted\to \;Data\;normalized\to \;spectrum\left[j\right]=\sum_{k=0}^{2^{j}-1}C_{j,k}^{2}$$

For the Haar wavelet, there would be $log_{2}\left(N\right)$ levels of decomposition. In the case of the classifier, each array of 15 elements was reduced to four levels using wavelet coefficients. In this case, the classifier would have input vectors with four elements.

In each facility, there were similar devices that introduced the same consumption patterns. For an adequate classification, the criterion of detecting the types of devices that existed by groups was taken. In addition, there were electrical appliances that could have different types of connection, depending on the type of programmed operation mode (washing machine, stove, dishwasher, etc.). Under these conditions, a prior automatic classification process was considered that helped a second supervised classification process to detect the type of connections that were to be detected. The steps to build the load classification model were:

1. Automatic cluster detection: Devices connections that existed in the installation were captured during representative periods of time. To perform a first analysis, an unsupervised classification method was used (kmeans). This first step was used to obtain information prior to making a supervised clustering decision. It is possible that many facilities do not know a priori what type of connections are produced. With this first analysis, the second step can be designed. In the test facility, 2000 connections were captured for three months. A first analysis based on the unsupervised method (*AffinityPropagation*) was performed automatically to detect the different clusters.
2. Feature extraction and clustering validation: In the initial step, the current types of loads were known and a first classification was made. The detected patterns must be validated in this phase to determine the clusters that should be detected in the classification process. Silhouette and cluster analysis were done in this phase. Model and training datasets clustered were the result.
3. Testing and learning validation. In this phase, we compared different classification methods such as *KNN, SVM, neural networks, etc.,* to decide which one to use. The model was tested and validated. The final result was a classification algorithm that would be installed and validated in an edge node.

**Figure 13:**
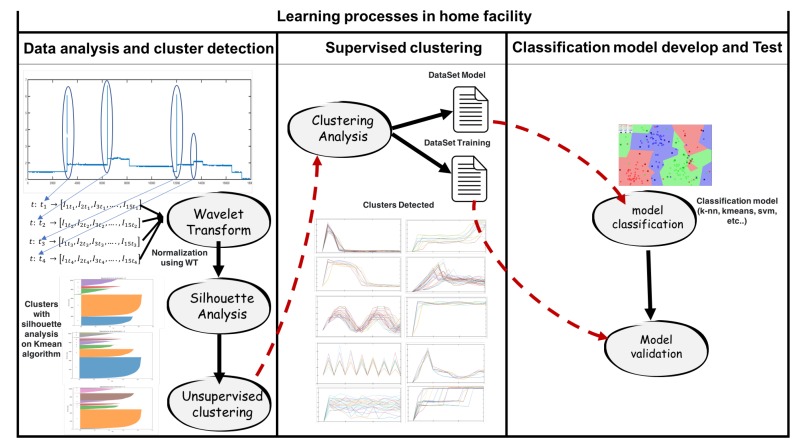
Learning process for classification in the home facility. Silhouette analysis and clusters detected in the experimental home show different phases of the learning process.

The process indicated above is shown in Figure 13.

**Figure 14:**
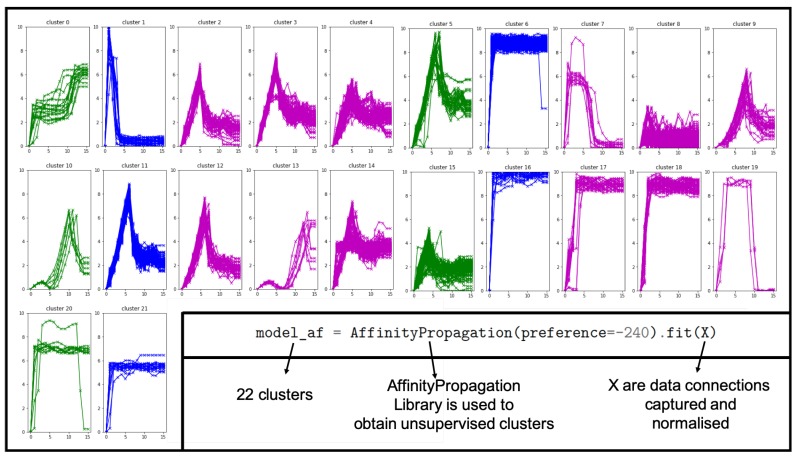
The result of using the unsupervised clustering method showed similar class groupings. They were not the definitive ones, because they had to be revised, modified, and validated. In facilities with many data, this first process is a good approximation to obtain an adequate clustering.

Once the model was validated, it needed to be implemented in an edge node device, connected to the consumption meter that captured the data. To implement the model, the Python programming language was used with a set of resources and libraries that allowed the implementation. In this process, Python language with the pandas, numpy, and sklearn libraries, among others, was used to obtain unsupervised clusters (Figure 14). The figure shows the result of applying the affinity method to 200 samples obtained by the electrical sensors. It can be appreciated that the function labeled different groupings that belonged to the same class and others that mixed different classes. For this reason, a supervision stage was introduced, in which an expert reviewed and modified those classes that were not well cataloged.

#### 5.4.2. Learning Process to Predict Power Consumption

**Figure 15:**
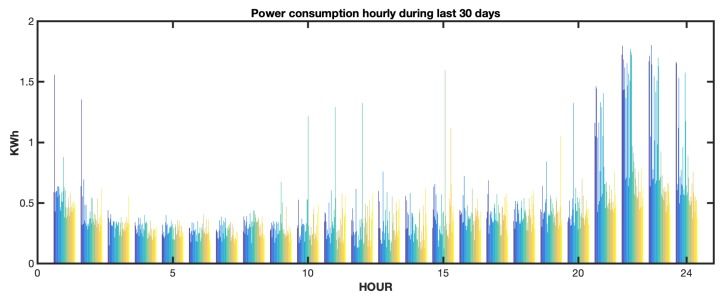
Power consumption dataset used to predict the consumption. For each hour of the day, on the x axis, the power consumptions during the last 30 days are shown. These data formed a dataset that was updated to be the input to the prediction algorithm.

**Figure 16:**
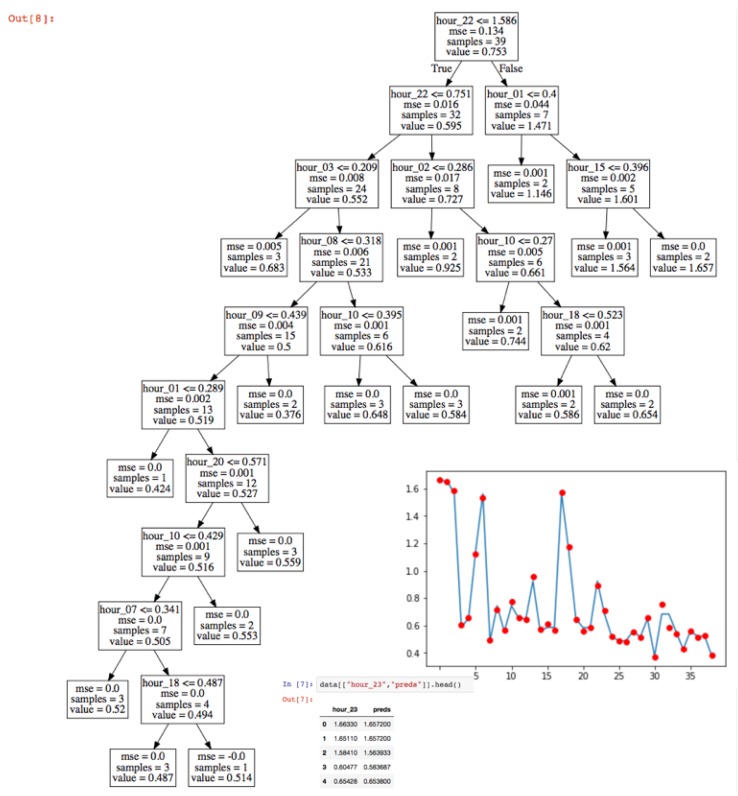
Regression tree obtained to predict power consumption hourly. The blue line represents the measured consumption and the red points the prediction made by the model.

The experimental work used an electrical consumption curve in a house with 110 m${}^{2}$ of useful area, with a distribution of 3 bedrooms, a living room, a kitchen, a gallery, 2 bathrooms, and 4 inhabitants. In this process, the consumption data of the last 30 days were captured, measured from hour to hour. These data constituted the input dataset to the regression model used. To create the predictor, a regression tree model was used. Figure 15 shows power consumption hourly during the last 30 days in a residential house captured by edge node processing. The sampling time in this process was t=1 h. Figure 16 shows the regression tree model obtained.

#### 5.4.3. Learning Process to Predict Renewable Power

In renewable power facilities, the model proposes a method to predict the energy that will be generated, knowing the capacity of the installation itself, the climate zone characteristics, the weather forecast, and the local climate sensors installed. As in the previous case, a learning process was designed. In this new case, a prediction algorithm based on IA paradigms was designed and implemented. Weather forecast and environmental sensors’ measurements (temperature, wind, etc.) were captured hourly. The climatic zone had data of the solar energy potential recorded for each season of the year. These data need to be accessible to perform the calculations. The sampling time in this process was t=1 h. This subsystem was designed according to a prediction based on:

- The power registered statistically for the area, measured every hour.
- The weather forecast of the area.
- The actual generation results captured in the installed solar panels, during the learning time.

With these data, an input dataset to a regression tree was obtained.

At this point, this subsystem was still in the learning phase. A heuristic that replaced the process designed to integrate the service in the operation of the smart grid was used. The heuristic was based on taking the statistical data of the area related to the generation, analyzing the weather forecast, and correcting said data according to the climate.

#### 5.4.4. Automatic Control Using Decision Rules

**Figure 17:**
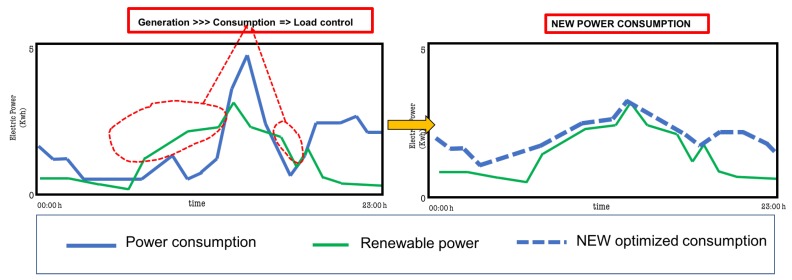
The figure on the left shows the forecast to be that there are hours in which generation will be higher than consumption. It is necessary to connect programmable loads to ensure that the consumption and generation are those of the figure on the right. If the generation is greater than the consumption, as happens at certain times, then the system must act by connecting automated loads. The figure on the right shows the new situation when the automatic activation of other loads occurs. The objective is to optimize the use of the generated energy and minimize the need for batteries (storage).

A decision tree calculated, hourly, different forecasts for the facility. The aim was to control power loads and renewable devices to optimize the use of renewable energy. The output of this decision tree was three possible scenarios. The result, every hour, was used as the input to the energy management system. If generation > consumption, electric devices (loads) needed to be connected to exploit energy. If consumption > generation, no control actions were taken. If consumption and generation can be different from the forecast time, control in real time must be implemented. The optimization process aim is shown in Figure 17. The sampling time in this process was t=1 h.

## 6. Experimental Work: Installation and Operation

**Figure 18:**
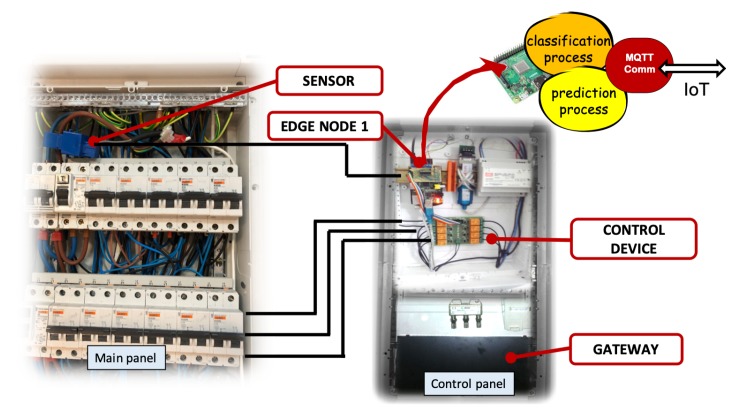
Experimental sub-system installed in the housing. The node can implement classification, prediction, and control algorithms proposed in the learning and operating phase.

At this level, the architecture was designed, as well as the functions and characteristics of the devices and nodes. In addition, classification and prediction models that ran on different nodes of the network were tested and validated. The operation processes integrated all the previous ones at different levels and put into operation another type of management, monitoring, and control services. This section shows the final algorithms implemented and their results in the experimental work. The designed services were installed in different edge nodes. One of them was installed in the main electrical box of the houses, another in the electric control panel of the renewable energy generation unit. All nodes communicated with each other using the IoT MQTT protocol. A cloud platform was used to obtain dashboard panels and event-based controls designed using cloud resources. The power consumption facility is shown in Figure 18. This subsystem allowed implementing algorithms with load classification services, consumption prediction, and connection control. The renewable subsystem was used to predict power generation.

### 6.1. Operation Process to Predict Power Generation and to Control

**Figure 19:**
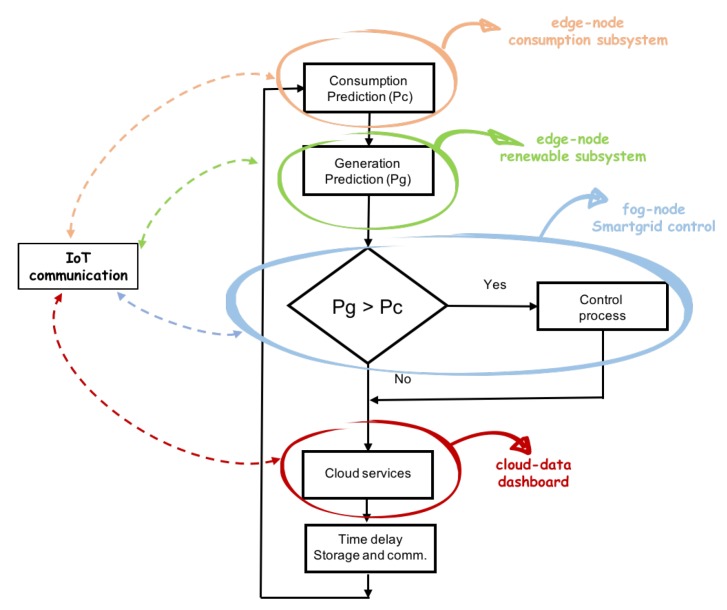
Processes installed in different devices integrated in the model. The figure shows their integration using a flow diagram. Each process designs and develops the applied services of detection, classification, or prediction in different nodes. These nodes can be edge or fog nodes, depending on the type of objective. The figure shows how the generation node and the consumption node provide the necessary data so that the load activation control process can act or not.

The system shown in Figure 19 represents the integration of different subsystems on different nodes. To predict renewable generation, a process took the statistical data of the area related to the generation, analyzing the weather forecast and correcting said data according to the climate. With this heuristic, the prediction of the generated power could be made, obtaining the necessary data to feed the control process and complete the optimized control process. Edge nodes calculated consumption and generation. The optimization and control process could be developed in fog or edge nodes that receive the data from generation and consumption units.

The aim was to control power loads and renewable devices to optimize the use of renewable energy. To control the installation, first of all, a decision tree that detected if generation was greater than consumption was developed. Then, if this event occurred, then a control algorithm decided if it could automatically connect a load to take advantage of that energy. These integrated services are shown in the Figure 19.

### 6.2. Operation Process to Classify Device Connections

**Figure 20:**
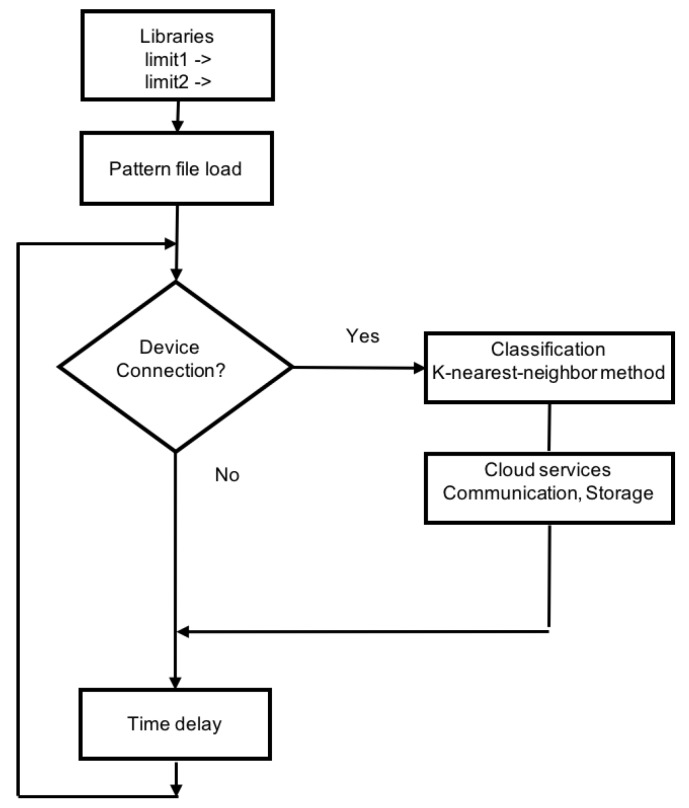
Device classification using K-nearest-neighbor.

The algorithm that detected and classified the connection type is displayed in Figure 20. The detected connection and its classification were stored in a file and were also sent to the UBIDOTSplatform, used as a cloud service. This algorithm was executed in an edge node, shown in Figure 18.

### 6.3. Operation Process to Predict Power Consumption

**Figure 21:**
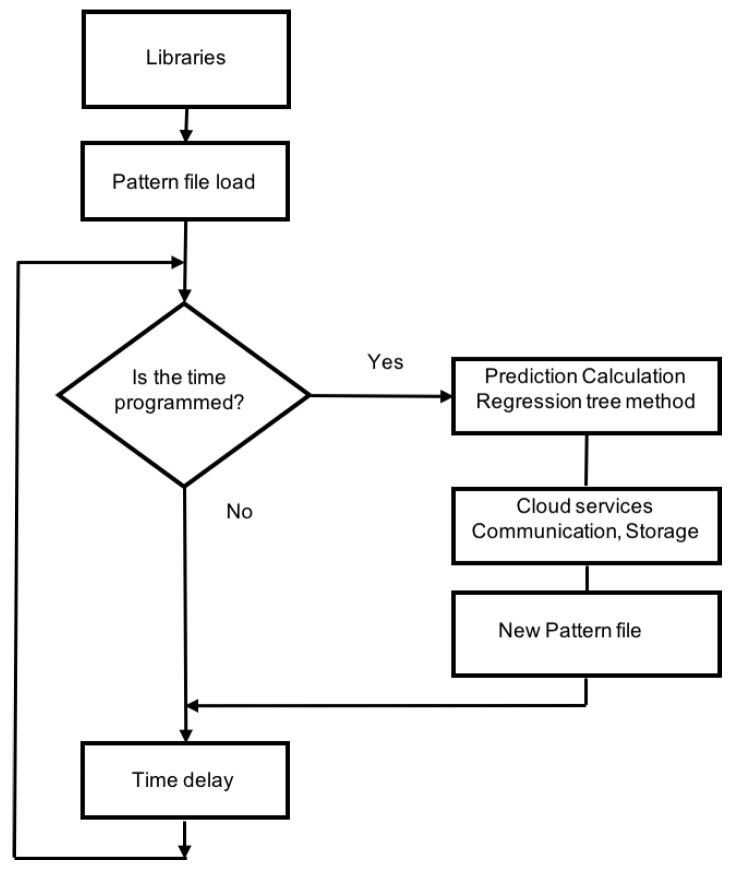
Power prediction algorithm using the regression tree paradigm.

Figure 21 shows how power prediction was developed on the edge node. Hourly, a new dataset with the last data capture was actualized. The algorithm was then executed with the updated dataset to predict consumption in the next hour. This algorithm was executed in an edge node, shown in Figure 18. A model based on regression trees was designed. Every hour, the previous consumptions were analyzed. These data were introduced in the model, and the model made a prediction.

## 7. Findings

**Figure 22:**
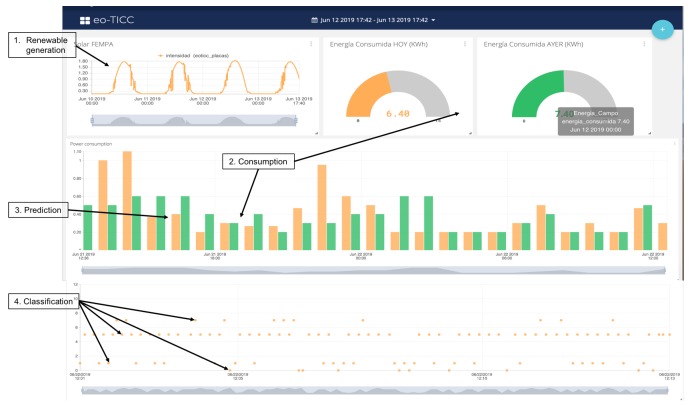
Dashboard results on the cloud platform: data captured by the generation (orange line in 1), consumption (green bar in 2), prediction (orange bar in 3), and classification (orange point in 4) algorithms implemented on edge nodes.

**Figure 23:**
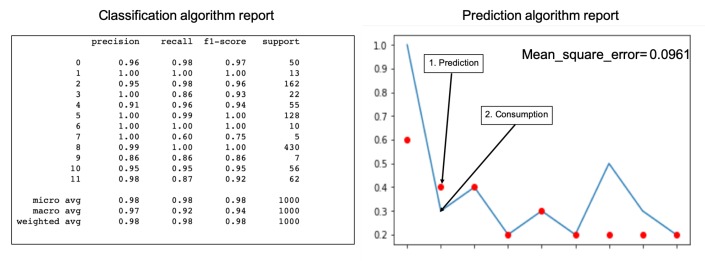
Classification and regression reports.

The major findings of the research were:

- The design and implementation of a set of algorithms in different nodes according to the defined architecture: The objective was to propose an architecture that was simple to implement and powerful to develop different services. The experimental results confirmed the objectives. Figure 19 shows the relationship of the different algorithms implemented and in operation in the experimental unit, and Figure 22 shows generation and consumption data on the cloud platform
- The design and implementation of a set of classification and prediction algorithms, based on artificial intelligence paradigms, which needed to be designed and implemented in the nodes of the architecture proposed: Figure 22 shows the results of the algorithms in a control panel. The consumption prediction section, in this figure, shows in orange the energy predicted by the prediction algorithm installed in the edge node that captured consumption data, in the first version of the prediction algorithm, the mean squared error is shown. In green, the real consumption data are shown.
- The classification module in Figure 22 shows the results of the load detection and classification algorithms implemented in different nodes. In Figure 23, a classification report is shown. The classification precision in this facility was greater than 86%. In Figure 23, a prediction report is shown. The mean squared error was 0.096, during the last three days (64 samples).
- The data generated by all the algorithms, installed in different nodes, are displayed in the same panel, managed by the fog node and by the cloud. It also shows the ease of the design and installation of the proposed architecture, as a result.
- Easy installation and operation as shown on installed devices and algorithms implemented: The economic costs were optimized using prediction algorithms and by minimizing storage use. If new batteries are not installed and programmable loads are managed, renewable units are easier to install and maintain. In the experimental unit, the solar panels installed amortized in less than four years with the energy produced, without installing batteries. All this was valid for installations connected to the grid, where the objective was to reduce energy dependence and generate savings of 30% with a small repayment period.
- With the embedded controller, in certain installations, the installation of batteries can be eliminated. The new controller activated loads automatically and distributed the generated energy. In these small facilities, the cost of amortization of the installation was less than five years. If there were no installation of batteries, the start-up and maintenance would be easier. This advantage would be applicable to homes connected to a grid, with the aim of reducing the energy cost.

## 8. Conclusions

A new model of integration for the development of energy management facilities has been proposed in this work. The model was based on the use of IoT communication protocols and artificial intelligence paradigms applied for classification, detection, prediction, and control of consumption and power generation systems. The artificial intelligence algorithms were deployed at two levels: the edge level and the fog level. Each level had different capacities and associated services. These two levels introduced a distributed architecture based on nodes. Each node could play a different role in terms of its computing and storage capabilities. All nodes communicated with each other using IoT communication protocols. The method made different levels of data processing interoperable with devices adapted to the services performed. In this work, algorithms were proposed to predict consumption and electrical generation, in addition to developing a load classification system. All these processes were installed in homes with renewable generation units. The result of these algorithms can be improved by investigating new paradigms of artificial intelligence that adapt better. New algorithms with new services can also be installed on the platform, in the same way as those performed in this work. The experimental results showed the benefits of the model: the ease of the design, installation, and operation, as well as optimized costs for the level of services developed. In future work, we plan the development of an API that integrates all the services developed in the intranet of the smart grid. This API will complete the interface that local and external systems will have to develop new services.

## Figures and Tables

**Table 1:** Device list and features.

Device	Hardware	Software-Comm.	Services
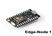	ARM Cortex-M4F 32-bit processor @ 64 MHz with 1 MB flash, 256 KB RAM [33] On-board additional 4 MB SPIflash and antenna 20 mixed signal GPIO Cost: $20–30	Real-time operating system (FreeRTOS) WiFi module, UART, I2C, SPI Open source design	Edge node Analog and digital sensors Communication data and control algorithms
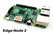	Broadcom BCM2837 64-bit ARMv7 Quad Core Processor running at 1.2 GHz [34] 1 GB LPDDR2SDRAM 40-pin extended GPIO Cost: $40–50	Free operating system based on Debian (Linux). BCM43143 WiFi on board, Bluetooth Low Energy (BLE) on board	Edge node Analog and digital sensor Control and AI operation algorithms
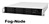	CPU Intel Core i5, 3 GHz Motherboard with chipset, 8 GB DDR3 256-GB SSD Cost: $400–1000	PC operating system WiFi, Bluetooth Learning processes, AI algorithms, smart grid management	Fog node Intranet and Internet communication, storage Learning processes, AI algorithms, smart grid management, analysis
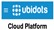	Cloud platform [30] Cost: $100–300 year	API REST Internet communication	Cloud services Dashboard, analysis, storage, events

## Abbreviations

The following abbreviations are used in this manuscript:

AI	Artificial Intelligence
IoT	Internet of Things
MQTT	Message Queuing Telemetry Transport
HTTPS	Hypertext Transfer Protocol Secure
HMI	Human–Machine Interface
M2M	Machine-to-Machine
API	Application Programming Interface
EMS	Energy Management Systems
HVAC	Heating Ventilation and Air Conditioning

## References

[1] Lin Y., Yu W., Zhang N., Yang X., Zhan H., Zhao W. (2017). A Survey on Internet of Things: Architecture, Enabling Technologies, Security and Privacy, and Applications. IEEE Internet Things J..

[2] Heđi I., Špeh I., Šarabok A. IoT network protocols comparison for the purpose of IoT constrained networks. Proceedings of the 2017 40th International Convention on Information and Communication Technology, Electronics and Microelectronics (MIPRO).

[3] Ferrández-Pastor F.J., García-Chamizo J.M., Nieto-Hidalgo M., Mora-Pascual J., Mora-Martínez J. (2018). Precision Agriculture Design Method Using a Distributed Computing Architecture on Internet of Things Context. Sensors.

[4] Ferrández-Pastor F.J., Mora-Mora H., Jimeno-Morenilla A., Volckaert B. (2018). Deployment of IoT Edge and Fog Computing Technologies to Develop Smart Building Services. Sustainability.

[5] Kaur K., Kaur K. A study of power management techniques for Internet of Things (IoT). Proceedings of the International Conference on Electrical, Electronics, and Optimization Techniques (ICEEOT).

[6] MQTT org. http://mqtt.org.

[7] Martinez C.M., Hu X., Cao D., Velenis E., Gao B., Wellers M. (2017). Energy Management in Plug-in Hybrid Electric Vehicles: Recent Progress and a Connected Vehicles Perspective. IEEE Trans. Veh. Technol..

[8] Sabri M.F.M., Danapalasingam K.A., Rahmat M.F. (2016). A review on hybrid electric vehicles architecture and energy management strategies. Renew. Sustain. Energy Rev..

[9] Beaudin M., Zareipour H. (2015). Home energy management systems: A review of modelling and complexity. Renew. Sustain. Energy Rev..

[10] Zhang D., Li S., Sun M., O’Neill Z. (2016). An Optimal and Learning-Based Demand Response and Home Energy Management System. IEEE Trans. Smart Grid.

[11] Minoli D., Sohraby K., Occhiogrosso B. (2017). IoT Considerations, Requirements, and Architectures for Smart Buildings—Energy Optimization and Next-Generation Building Management Systems. IEEE Internet Things J..

[12] Farrokhifar M., Momayyezi F., Sadoogi N., Safari A. (2018). Real-time based approach for intelligent building energy management using dynamic price policies. Sustain. Cities Soc..

[13] Kuehn P.J., Mashaly M.E. (2015). Automatic energy efficiency management of data center resources by load-dependent server activation and sleep modes. Ad Hoc Netw..

[14] Hameed A., Khoshkbarforoushha A., Ranjan R. (2016). A survey and taxonomy on energy efficient resource allocation techniques for cloud computing systems. Computing.

[15] Calvillo C.F., Sánchez-Miralles A., Villar J. (2016). Energy management and planning in smart cities. Renew. Sustain. Energy Rev..

[16] Liu Y., Yang C., Jiang L., Xie S., Zhang Y. (2019). Intelligent Edge Computing for IoT-Based Energy Management in Smart Cities. IEEE Netw..

[17] Olatomiwa L., Mekhilef S., Ismail M.S., Moghavvemi M. (2016). Energy management strategies in hybrid renewable energy systems: A review. Renew. Sustain. Energy Rev..

[18] Liu Y., Fieldsend J.E., Min G. (2017). A Framework of Fog Computing: Architecture, Challenges, and Optimization. IEEE Access.

[19] Khan M.R.B., Jidin R., Pasupuleti J. (2016). Multi-agent based distributed control architecture for microgrid energy management and optimization. Energy Convers. Manag..

[20] Faruque M.A.A., Vatanparvar K. (2016). Energy Management-as-a-Service Over Fog Computing Platform. IEEE Internet Things J..

[21] Moghaddam M.H.Y., Leon-Garcia A. (2018). A Fog-Based Internet of Energy Architecture for Transactive Energy Management Systems. IEEE Internet Things J..

[22] Marzband M., Yousefnejad E., Sumper A., Domínguez-García J.L. (2016). Real time experimental implementation of optimum energy management system in standalone Microgrid by using multi-layer ant colony optimization. Int. J. Electr. Power Energy Syst..

[23] Marino D.L., Amarasinghe K., Manic M. Building Energy Load Forecasting using Deep Neural Networks. Proceedings of the IECON 2016 42nd Annual Conference of the IEEE Industrial Electronics Society.

[24] Wang H., Huang J. (2018). Incentivizing Energy Trading for Interconnected Microgrids. IEEE Trans. Smart Grid.

[25] Wang H., Huang J. (2016). Cooperative Planning of Renewable Generations for Interconnected Microgrids. IEEE Trans. Smart Grid.

[26] Nunna H.S.V.S.K., Doolla S. (2012). Demand response in smart distribution system with multiple microgrids. IEEE Trans. Smart Grid.

[27] Gregoratti D., Matamoros J. (2015). Distributed energy trading: The multiple-microgrid case. IEEE Trans. Ind. Electron..

[28] Amazon IoT Cloud Platform. https://aws.amazon.com/es/iot-core/.

[29] Google IoT Cloud Platform. https://cloud.google.com/iot-core/?hl=es.

[30] Ubidots IoT Cloud Platform. https://ubidots.com.

[31] Microsoft IoT Cloud Platform. https://azure.microsoft.com/es-es/overview/iot/.

[32] Dark Data Inform. https://www.gartner.com/it-glossary/dark-data.

[33] Embedded Device Controller Edge-Node 1. https://www.particle.io.

[34] Embedded Device Controller Edge-Node 2. https://www.raspberrypi.org.

[35] Subramani P., Sahu R., Verma S. (2006). Feature selection using Haar wavelet power spectrum. BMC Bioinform..

